# Impact of Smart Wearable Devices on Health and Health Inequality Among Older Adults: Evidence from China

**DOI:** 10.3390/healthcare14060813

**Published:** 2026-03-22

**Authors:** Xiaohui Wang, Yaqi Li, Wenlong Lou

**Affiliations:** 1 School of Public Administration, Yanshan University, Qinhuangdao 066004, China; wxiaohui@ysu.edu.cn (X.W.); louwenlong@ysu.edu.cn (W.L.); 2 Beijing-Tianjin-Hebei Cooperative Development Management Innovation Research Centre, Qinhuangdao 066004, China

**Keywords:** smart wearable devices, older adults, health, health inequality, smart care for older adults

## Abstract

**Background**: As China enters the digital era and actively promotes an active aging strategy, smart wearable devices have become increasingly prevalent among older adults; however, their impact on health inequality remains unclear. This study investigates the association between smart wearable devices and health, as well as health inequality, among Chinese older adults, and further examines the mediating roles of joy of living and social participation. **Methods**: Data were derived from two waves (2018 and 2020) of the China Longitudinal Aging Social Survey (CLASS), with a final sample of 7098 adults aged 60 and above. A two-way fixed-effects model, propensity score matching–difference-in-differences (PSM-DID) approach, and mediation analysis were employed. **Results**: Smart wearable devices were significantly positively associated with both health and health inequality among older adults in China. Mediation analysis revealed that joy of living and social participation played an intermediary role. **Conclusions**: This study provides preliminary evidence that smart wearable devices are associated with health and health inequality among Chinese older adults. Policy efforts should focus on developing more user-friendly devices, promoting digital literacy among older adults, and supporting disadvantaged groups. Furthermore, the mediating effects suggest that fostering joy of living and encouraging active social participation may serve as effective pathways to improve health.

## 1. Introduction

As the global trend of population aging intensifies, the health of older adults and associated health inequalities have become a major social concern. According to the World Health Organization’s Decade of Action for Healthy Ageing 2020–2030 report, the proportion of the global population aged 65 and over is expected to increase from 9% in 2019 to 16% by 2050, with particularly pronounced growth in China [1]. Data from the Seventh National Population Census indicates that China’s population aged 60 and above has reached 264 million, accounting for 18.7% of the total population. Among them, approximately 40 million are disabled or partially disabled, and 78% of those aged 65 and over suffer from at least one chronic disease [2]. More concerning are the marked disparities across geographic regions, economic levels, and social backgrounds in access to medical resources, health literacy, chronic disease management, and rehabilitation services. These disparities have rendered health and health inequality among older adults an increasingly pressing issue [3].

As technology and health become increasingly integrated, the rapid spread of the Internet is reaching all segments of society at an unprecedented pace, ushering older adults into a new era of digital life. According to statistics, as of June 2024, China had gained 7.42 million new Internet users, with adults aged 60 and above accounting for 20.8% of this growth [4], which highlights the enthusiasm of older adults in embracing the digital age. With the continued proliferation of Internet technology, smart wearable devices are playing an increasingly vital role in the lives of older adults [5]. Reports indicate that China’s smart aging market exceeded 6 trillion yuan in 2023, with smart wearable devices alone reaching a market size of 93.47 billion yuan, establishing them as one of the dominant segments in the smart aging industry [6,7]. Health capital theory conceptualizes health as a form of capital that can be accumulated and enhanced through proactive interventions, suggesting that individuals can increase their health capital stock and thereby improve their health status through health monitoring, behavioral adjustments, and investment in health resources [8]. Smart wearable devices offer a unique combination of user-friendly design, portability, and real-time health monitoring capabilities, enabling timely alerts for abnormal health indicators. The above features effectively address the urgent healthcare and emergency assistance needs of older adults, making them valuable tools for health management. On the one hand, these devices can monitor daily activity levels and track dietary and medication habits, helping users maintain a healthy lifestyle [9]. On the other hand, most smart wearable devices are equipped with emergency response functions that play a crucial role in ensuring the safety of older adults [10,11]. Among the various smart wearable devices, smart bracelets are particularly well-regarded for their lightweight design, ease of use, and comprehensive functionality [12,13], which not only continuously monitor key health indicators and provide instant feedback but also encourage physical activity through step counting and goal-setting features, fostering a positive attitude toward life [14]. The application value of smart wearable devices in promoting older adult health has begun to emerge. From the perspective of research frontiers, a recent study by Cai et al. (2025) has further expanded the exploration of wearable devices in health management to enhance their practical value in population health promotion [15].

The rapid proliferation of smart wearable devices has positioned them as important technological tools for improving the health of older adults in the digital age. However, the relationship between digital technology and health inequality among older adults has long been a subject of academic debate. Some scholars argue that the widespread use of the Internet and digital information technologies is associated with significant “digital dividends,” which may benefit users’ physical and mental health and could potentially contribute to reducing health inequality [16,17]. In contrast, others contend that some older adults struggle to adapt appropriately to the digital environment. Excessive Internet use and over-reliance on online social media may be linked to adverse physical and mental health among older users, showing a significant positive association with increased health inequality [18]. Smart wearable devices are not uniformly adopted among older adults, which has drawn scholarly attention to a core question: while these devices may influence the health of older adults, could they also exacerbate health inequality due to group differences in access and usage capability? Digital divide theory focuses on disparities in the distribution and use of digital technologies across different groups. It suggests that individual heterogeneity in socioeconomic status, education level, and digital literacy leads to significant stratification in access to, exposure to, and effective use of digital technologies. Such technological inequality may ultimately translate into gaps in welfare and developmental opportunities [19].

Amid China’s rapidly aging population, active aging has emerged as a core strategy for addressing aging-related challenges and promoting healthy aging, giving rise to the active aging framework. This framework moves beyond a narrow focus on physical health to emphasize non-physiological factors such as social participation and subjective well-being as essential to achieving healthy aging. It advocates enhancing older adults’ joy of living and expanding their opportunities for social engagement to support higher-quality development in later life [20]. The impact of smart wearable devices on the health and health inequality of older adults is not solely realized through technical functions such as health monitoring or emergency alerts. Instead, it may gradually unfold through the mediating pathways of joy of living and social participation. The indirect pathways through which smart wearable devices influence health are likely to operate differently across older adult subgroups. For some users, the engaging incentives and real-time feedback may enhance the joy of living, while for others, data-sharing features may facilitate social interaction and broaden participation. However, the extent to which individuals benefit depends critically on factors such as digital literacy, socioeconomic status, and usage context. This leads to heterogeneous health gains across groups and potentially widens health disparities.

**Figure 1:**
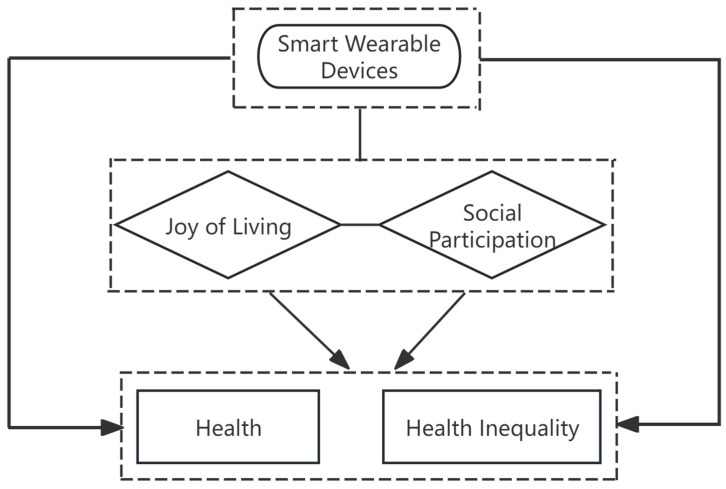
Research framework.

Health capital theory clarifies the underlying logic through which devices may affect the health of older adults, the active aging framework highlights the key roles of joy of living and social participation, and the digital divide theory reveals the roots of group differences in the use of smart wearable devices. Based on this integrated theoretical framework (see Figure 1), the present study investigates the relationship between smart wearable devices and health outcomes and health inequality among older adults using Chinese data. This research also explores the underlying mechanisms through which smart wearable devices influence health and health inequality in this population. The findings aim to provide new insights for the field and offer a scientific basis for policies related to resource allocation and the promotion of regional health equity.

## 2. Materials and Methods

### 2.1. Data

The microdata used in this study are drawn from the China Longitudinal Aging Social Survey (CLASS). Conducted by the China Survey and Data Center at Renmin University of China, CLASS is a nationally representative longitudinal survey targeting adults aged 60 and above. The first baseline survey was conducted in 2014, followed by three waves of follow-up surveys in 2016, 2018, and 2020. The survey employs a stratified multi-stage probability sampling design, covering 23 provinces (including autonomous regions and municipalities directly under the central government), 29 prefecture-level cities, 7 prefectures, 30 autonomous prefectures, and 3 leagues across China. The CLASS database provides comprehensive information on older adults, including basic demographics, family background, physical health, and home environment, and a substantial body of existing research has utilized CLASS data to investigate health and social participation in aging populations, further supporting its suitability for this study [21,22]. Since the CLASS only began collecting information on smart bracelets and related variables in 2018, this study uses data from the 2018 and 2020 waves. Based on the merged data, a total of 11,418 respondents were identified in 2018, of whom 9164 were successfully re-interviewed in 2020, yielding a sample attrition rate of 19.74%. After excluding observations with missing values on key variables, the final analytic sample comprised 14,196 observations (7098 people per year).

### 2.2. Measures

#### 2.2.1. Dependent Variables

(1) Self-rated health. Self-rated health (SRH) has been shown in the literature to be a reliable indicator of individuals’ actual health status [23,24]. Accordingly, this study assesses the health of older adults using responses to the following question from the health and related services section of CLASS: “How do you feel about your current physical health status?” Responses are coded on a five-point scale ranging from 1 (“very unhealthy”) to 5 (“very healthy”), with higher scores reflecting better perceived health.

(2) Health Inequality. Various methods exist for measuring health inequality, including the relative deprivation index, concentration index, range method, Lorenz curve, and Gini coefficient. Among these, the relative deprivation index has been widely adopted in empirical research [25]. Kakwani (1984) [26] argues that an individual’s sense of deprivation arises from comparisons with others. When individuals perceive themselves to be in a disadvantaged position, they experience relative deprivation. He operationalized this sociological concept into a quantifiable index, the core logic of which is that the degree of relative deprivation is jointly determined by two factors: the proportion of people with better health status, and the average health gap between the individual and those who are better off. Grounded in relative deprivation theory, this approach posits that the degree of health inequality intensifies as older adults in disadvantaged health positions experience deeper relative deprivation. Accordingly, this study employs the relative deprivation index to quantify the extent of health inequality among older adults. The index is calculated as follows:

(1) $$\mathrm{Health\_RD}(y_{j},y_{i})=\frac{1}{{nu}_{Y}}\sum_{j=i+1}^{n}(y_{j}-y_{i})=r_{y_{i}}^{+}\left[\frac{\left(u_{y_{i}}^{+}-y_{i}\right)}{u_{Y}}\right]$$

In Equation (1), Health_RD (health relative deprivation index) represents the health status of older individuals within the group, with a value range of [0, 1]. A value of 0 indicates that an individual’s health status is equal to or better than that of all other older adults, while values closer to 1 reflect deeper relative deprivation and more severe health inequality within the group. $y_{i}$ represents the health status of individual i, and $u_{Y}$ represents the average health status of individuals in group Y. $r_{y_{i}}^{+}$ represents the proportion of individuals in group Y with health levels better than $y_{i}$, and $u_{y_{i}}^{+}$ represents the average health status of individuals in group Y with health levels exceeding $y_{i}$.

#### 2.2.2. Independent Variables

Characterized by their lightweight design, multifunctionality, and real-time health monitoring capabilities, smart bracelets are among the most popular smart wearable devices for older adults. Due to data availability constraints, this study uses smart bracelets as a representative indicator of smart wearable device ownership in this population. Ownership is assessed based on responses to the following question from the older care planning and social support section of CLASS: “Do you have a smart bracelet?” Responses are coded as 0 for “no” and 1 for “yes.”

#### 2.2.3. Mediating Variables

This study examines the joy of living and social participation as mediating variables. The joy of living is measured using responses to the following psychological perception question from the CLASS questionnaire: “Did you feel that there was a lot of fun in life in the past week?” Responses are coded as 1 for “often,” 2 for “sometimes,” and 3 for “never.”

Social participation is assessed based on responses to seven items from the pension planning and social support section of the CLASS questionnaire, which asks: “What is the frequency of your participation in the following activities?” The activities include mediating neighborhood disputes, providing volunteer services using professional skills, and caring about the education of the next generation. For each activity, responses are coded on a five-point scale: 0 for “did not participate,” 1 for “several times a year,” 2 for “at least once a month,” 3 for “at least once a week,” and 4 for “almost every day.” The scores across all seven items are summed to create a composite measure, with higher total scores indicating greater levels of social participation.

#### 2.2.4. Controlled Variables

To minimize omitted variable bias, this study includes a set of control variables in the regression models, encompassing both personal and social characteristics. Personal characteristics comprise gender, age, marital status, education level, number of children, living arrangement, household registration (hukou), and household income. Social characteristics include social security benefits, frequency of internet use, and financial support from children. In addition, province-level dummy variables are included to account for regional heterogeneity.

#### 2.2.5. Analytical Strategies

Descriptive analyses were first conducted for all variables. A two-way fixed-effects model was then employed to examine the associations between smart wearable device use and health, as well as health inequality, among older adults, and then to test the mediating roles of joy of living and social participation. To assess the robustness of the findings, propensity score matching combined with a difference-in-differences approach (PSM-DID) was applied.

(1) Two-way fixed-effects model

To estimate the effects of smart wearable devices on health and health inequality among older adults, this study employs a two-way fixed-effects model. The baseline regression specifications are as follows:

(2) $${Health}_{ipt}=\alpha_{0}+\beta_{1}{SW}_{ipt}+\beta_{2}{X_{ipt}+y}_{p}+\delta_{i}+\theta_{t}+\epsilon_{ipt}$$

(3) $${Health\;Inequality}_{ipt}=\varphi_{0}+\tau_{1}{SW}_{ipt}+\tau_{2}{\mathrm{Health\_RD}}_{ipt}+\tau_{3}X_{ipt}+y_{p}+\delta_{i}+\theta_{t}+\epsilon_{ipt}$$

In Equations (2) and (3), ${Health}_{ipt}$ denotes the health status of older adult i in province p at time t. ${Health\;Inequality}_{ipt}$ represents the corresponding level of health inequality. ${\mathrm{Health\_RD}}_{ipt}$ is the relative deprivation index for individual i at time t, ${SW}_{ipt}$ (Smart Wearable) is a binary indicator of whether the individual uses a smart wearable device. $X_{ipt}$ is a vector of control variables. $y_{p}$ represents province fixed-effects, $\theta_{t}$ denotes time fixed-effects, and $\delta_{i}$ captures individual fixed-effects. $\epsilon_{ipt}$ is the idiosyncratic error term.

(2) Propensity score matching and difference in difference method (PSM-DID)

To test the robustness of the main findings, this study employs a propensity score matching combined with a difference-in-differences approach (PSM-DID). The model specification is as follows:

(4) $$Logit(P(T_{i}=1|Z_{i}))=\theta_{0}+\theta_{1}Z_{i1}+\theta_{2}Z_{i2}+\ldots \ldots +\theta_{k}Z_{ik}$$

The propensity score is estimated using a Logit model, which predicts the probability that an older adult receives the treatment, in this case, using a smart sleep monitor. This probability is referred to as the propensity score. The model includes all covariates that may influence treatment assignment. In Equation (4), Ti denotes the treatment indicator (1 for treated, 0 for untreated), Zi is a vector of individual characteristics, and θ represents the vector of parameters to be estimated.

(5) $$Y_{it}=\alpha +\delta_{1}T_{i}+\delta_{2}{Post}_{t}+\delta_{3}(T_{i}\times {Post}_{t})+y\mu_{i}+\in_{it}$$

The difference-in-differences (DID) estimator identifies the treatment effect by comparing the average change in outcomes between the treatment and control groups before and after the intervention. In Equation (5), $Y_{it}$ denotes the health status or health inequality of individual i at time t. The variable $T_{i}$ indicates treatment assignment (1 for treated, 0 for control), and ${Post}_{t}$ is a time indicator equal to 1 for the post-treatment period (2020) and 0 for the pre-treatment period (2018). The interaction term $T_{i}\times {Post}_{t}$ captures the differential change attributable to the treatment, with its coefficient $\delta_{3}$ representing the treatment effect. The vector $\mu_{i}$ includes individual-level control variables, and $\epsilon_{ipt}$ is the idiosyncratic error term.

## 3. Results

### 3.1. Descriptive Statistics

Table 1 presents the descriptive statistics for all variables included in the analysis. Between 2018 and 2020, the proportion of older adults owning smart wearable devices increased notably, rising from 3.1% to 7.9%—a growth of 4.8 percentage points. Over the same period, the overall health status of older adults remained relatively stable, while health inequality showed a modest improvement, decreasing from 0.08 in 2018 to 0.02 in 2020.

### 3.2. Regression Analysis

A two-way fixed-effects model was estimated to examine the association between smart wearable devices and health, as well as health inequality, among older adults. The regression results are presented in Table 2. Models (1) and (3) only include the key independent variable, smart wearable devices, and reveal a statistically significant positive association with both health and health inequality at the 1% level. Specifically, compared to non-users, older adults who use smart wearable devices report better health, yet this group also exhibits higher levels of health inequality. After incorporating a comprehensive set of personal and social characteristics as control variables in Models (2) and (4), the positive associations remain statistically significant at the 1% level.

### 3.3. Robustness Testing

#### 3.3.1. Estimation Based on Propensity Score Matching with Difference-in-Differences (PSM-DID)

Older adults’ health status and health inequality are shaped by a combination of personal characteristics and social factors rather than being randomly determined. To mitigate potential selection bias and endogeneity concerns, this study employs a propensity score matching combined with a difference-in-differences approach (PSM-DID) in the robustness analysis, aiming to strengthen the identification of causal relationships.

First, a common support test was conducted to ensure comparability between the treatment and control groups. Propensity scores were estimated using a Logit model that included covariates potentially influencing both smart wearable devices and health. The matching variables—all measured at baseline (2018)—comprised age, gender, marital status, number of children, living arrangement, household registration (hukou), education level, household income, social security benefits, financial support from children, and frequency of internet use. The test results indicate substantial overlap in the propensity score distributions between the two groups, with 98.56% of the sample (13,992 observations) falling within the common support region. Specifically, 783 treated and 13,209 control observations were retained within the common support, while 204 observations (1.44%) lying outside this region were excluded to ensure the reliability of the treatment effect estimates.

**Figure 2:**
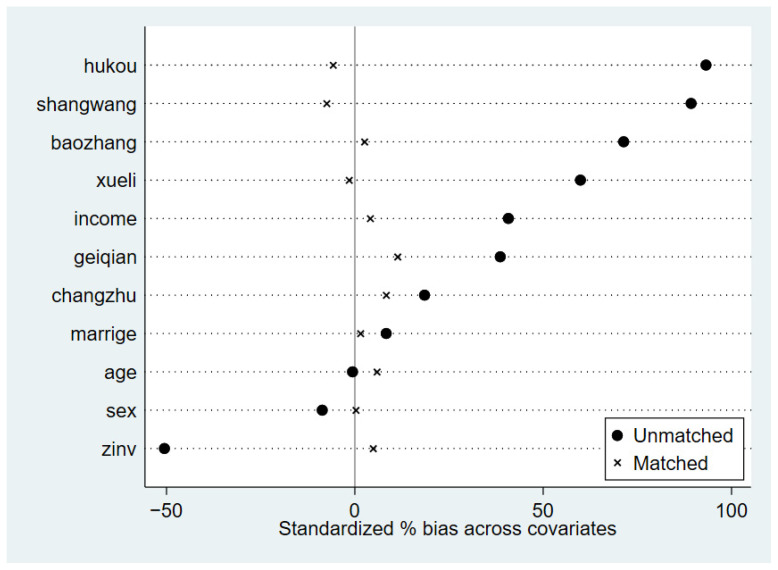
Standardized biases of covariates before and after matching.

Next, a propensity score matching (PSM) balance test was performed, with health status used as an illustrative example (see Figure 2). The results indicate that before matching, the standardized biases of the covariates were relatively large; after matching, these biases were substantially reduced, all falling below the recommended threshold of 10%. Building on the successful implementation of the PSM procedure, the study further assessed the balance of covariates within the PSM-DID framework. As shown in Table 3, after matching, the *t*-tests failed to reject the null hypothesis of no systematic differences between the treatment and control groups. These findings provide strong evidence that the PSM-DID approach effectively mitigates observable bias in the sample, thereby enhancing the reliability and robustness of the estimation results.

#### 3.3.2. Replace the Dependent Variables

The World Health Organization (WHO) defines health as “a state of complete physical, mental, and social well-being and not merely the absence of disease or infirmity” [27]. Drawing on this holistic perspective, this study further tests the robustness of the main findings by replacing the dependent variables with social adaptability and its associated inequality.

As shown in Table 4, the positive association between smart wearable devices and older adults’ social adaptability and its inequality remained statistically significant at the 5% level, which is consistent with the baseline regression results and further confirms the robustness of the findings.

### 3.4. Endogeneity Treatment

Potential endogeneity may arise from reverse causality, as healthier older adults could be more inclined to use smart wearable devices. To address this, and considering the limitations of questionnaire data availability, this study follows the approach of Fang and Wen [28] by employing “whether the respondent’s residence has internet signal” as an instrumental variable. Given that most smart wearable devices require an internet connection to function, signal availability directly influences the likelihood of use, yet it is unlikely to be directly correlated with older adults’ health or health inequality.

Table 5 presents the instrumental variable regression results. The instrument passes both weak identification and overidentification tests, confirming its validity. After accounting for endogeneity, the positive associations between smart wearable devices and health, as well as health inequality, among older adults, remain statistically significant at the 1% level. The direction and magnitude of these estimates are consistent with the baseline results, providing further support for the conclusion that while smart wearable devices are associated with improved health, they may also be linked to increased health inequality in this population.

### 3.5. Mediation Analysis

After establishing the associations between smart wearable devices and health, as well as health inequality, among older adults, this study further examines the underlying mechanisms by drawing on the mediation analysis framework proposed by Wen and Ye [29]. Specifically, beyond the devices’ health monitoring and disease prevention functions, the mediating roles of joy of living and social participation are investigated.

Models (1) and (2) indicate that among older adults using smart wearable devices, higher levels of joy of living and greater social participation are associated with better health at the 1% and 5% significance levels, respectively. In contrast, Models (3) and (4) suggest that while these improvements may enhance quality of life, they are also associated with increased health inequality among older adults at the 1% significance level. The full results of the mediation analysis are presented in Table 6.

### 3.6. Heterogeneity Analysis

The baseline regression results indicate that smart wearable devices are significantly positively associated with both health and health inequality among older adults. To further explore heterogeneity, subgroup analyses were conducted by income, education, urban–rural residence, and gender, with results presented in Table 7.

For education, urban-rural residence, and gender, smart wearable device use is significantly positively associated with both health and health inequality across all subgroups at the 1% or 5% significance level, with relatively comparable coefficient magnitudes. However, notable heterogeneity emerges by income: significant positive associations are observed only among high-income older adults at the 1% level, while no significant associations are found for their low-income counterparts.

## 4. Discussion

The results indicate that smart wearable devices are significantly positively associated with health, as well as health inequality, among older adults. These findings align with prior research examining the relationship between Internet technology and health outcomes and health inequality in this population [30,31,32], and further support the conceptualization of smart wearable devices as an extension of Internet-based technologies.

Beyond their functions in health monitoring and disease prevention, smart wearable devices have also emerged as important tools for enhancing the joy of living and promoting social participation among older adults [33,34]. The mediation analysis confirms that joy of living and social participation serve as key mediating variables in the relationship between smart wearable devices and health, as well as health inequality. By facilitating engagement with daily life, wearable devices enable older adults to discover and experience greater joy, which not only helps reduce stress, build psychological resilience, and improve subjective well-being but also encourages social interaction, strengthens interpersonal relationships, and stimulates creativity and learning [35].

Moreover, older adults who use smart wearable devices tend to be more socially engaged than non-users. By integrating personalized entertainment features and strengthening social connections, these devices enrich the daily lives of older adults [36]. Their application also supports the development of telemedicine monitoring and personalized companion services [37], enabling older adults to remain active and healthy in later life while further enhancing their social participation. Social engagement, in turn, provides older adults with additional social support, and the diversity of social relationships helps shape health-related behaviors and lifestyle patterns [38], contributing to significant positive effects on health [39].

The reason why smart wearable devices contribute to health inequality among older adults lies in differences in device access, usage patterns, and the extent to which they benefit from using such devices [40,41]. In access, higher-income seniors are more likely to own advanced devices, while disadvantaged groups often face financial and environmental barriers. Even when obtained, their devices tend to be basic models with limited functionality. In usage, many older adults are confined to basic features like step counting, unable to use core functions such as health monitoring or risk alerts, which limits the devices’ health potential. In outcomes, those with better access and digital literacy benefit more from health monitoring and risk prevention, contributing to improved health. In contrast, disadvantaged groups gain less, further widening the health gap.

As China advances the “Healthy China 2030” strategy and promotes active aging, smart wearable devices have become a key link between technological innovation and health management for older adults, with their value increasingly recognized. Based on this, the following policy recommendations are proposed: First, optimize device supply by encouraging enterprises to focus on the core health needs of older adults and develop wearable devices that are user-friendly and offer accurate monitoring, with enhanced functions such as heart rate and blood pressure tracking and health alerts. Governments can reduce production and sales costs through tax incentives and subsidies, thereby improving device accessibility for older adults. Second, narrow regional and group disparities by increasing support for remote and economically disadvantaged areas, upgrading network infrastructure to ensure proper device usage, and providing targeted subsidies for low-income older adults. Efforts should also be made to improve health data sharing between urban and rural areas to promote a more equitable distribution of healthcare resources. Third, enhance usage capacity by providing training on device operation and health data interpretation to improve digital literacy among older adults. Simultaneously, encourage enterprises to enrich interactive features, such as social engagement and health check-ins, to boost user motivation, enabling older adults to fully realize the health benefits of smart wearables and ultimately reduce health disparities.

This study has several limitations. First, because the CLASS project began collecting information on smart wearable devices only in 2018, the analysis is restricted to two waves of data (2018 and 2020), limiting the ability to examine long-term effects. Second, using smart bracelets as the sole proxy for smart wearable devices may not capture the full range of device types and functionalities available to older adults. Third, due to data constraints, health status is measured using self-rated health. While this indicator is widely used in aging research, it is subject to reporting bias. Moreover, despite efforts to address endogeneity, potential threats such as omitted variable bias, reverse causality, and self-selection bias cannot be entirely ruled out. Future research could employ multi-wave panel data to examine long-term dynamic effects, incorporate a broader range of device types to capture heterogeneous effects, combine objective health measures with self-rated health to reduce measurement bias, and apply more rigorous causal inference methods to mitigate endogeneity concerns.

## Figures and Tables

**Table 1:** Sample characteristics.

Variables	Definitions	2018	2020
		(Mean (SD))/ (Frequency (Percent))	(Mean (SD))/ (Frequency (Percent))
Self-rated health	Level 1 = 1	148 (2.09%)	161 (2.27%)
	Level 2 = 2	877 (12.36%)	900 (12.68%)
	Level 3 = 3	2705 (38.11%)	2627 (37.01%)
	Level 4 = 4	2844 (40.07%)	2865 (40.36%)
	Level 5 = 5	524 (7.38%)	545 (7.68%)
Health inequality	Relative deprivation index	0.08 (0.97)	0.02 (0.98)
Smart wearable devices	No = 0	0.03 (0.17)	0.08 (0.27)
	Yes = 1		
Age	Continuous variable	69.74 (6.25)	71.74 (6.25)
Gender	Female = 0	3515 (49.52%)	3510 (49.45%)
	Male = 1	3583 (50.48%)	3588 (50.55%)
Marriage	No = 0	1928 (27.16%)	1775 (25.01%)
	Yes = 1	5170 (72.84%)	5323 (74.99%)
Children’s number	Continuous variable	2.40 (1.29)	2.38 (1.30)
Residence	Live alone = 0	817 (11.51%)	703 (9.90%)
	Else = 1	6281 (88.49%)	6395 (90.10%)
Household registration	Rural = 0	3998 (56.33%)	4050 (57.06%)
	Urban = 1	3100 (43.67%)	3048 (42.94%)
Education level	Illiterate = 1	1682 (23.70%)	1676 (23.61%)
	Literacy class = 2	285 (4.00%)	303 (4.27%)
	Primary school = 3	2603 (36.70%)	2603 (36.67%)
	Junior high school = 4	1770 (24.90%)	1758 (24.77%)
	High school = 5	597 (8.40%)	597 (8.41%)
	Junior college = 6	137 (1.90%)	137 (1.93%)
	Undergraduate and above = 7	24 (0.30%)	24 (0.34%)
Household income level (In logarithm)	Continuous variable	10.23 (1.75)	10.49 (0.53)
Social Security (In logarithm)	Continuous variable	6.17 (2.94)	5.01 (2.49)
Economic supply	Continuous variable	3379.36 (4208.03)	3151.61 (3770.49)
Internet usage	Never been online = 1	5629 (79.30%)	5278 (74.36%)
	Several times a year = 2	49 (0.69%)	44 (0.62%)
	At least once a month = 3	62 (0.87%)	64 (0.90%)
	At least once a week = 4	334 (4.70%)	421 (5.93%)
	Everyday = 5	1024 (14.43%)	1291 (18.19%)
Province fixed-effects	Dummy variable	controlled	controlled

**Table 2:** Baseline regression results.

Variables	(1) Health	(2) Health	(3) Health Inequality	(4) Health Inequality
Smart wearable devices	0.410 *** (0.048)	0.362 *** (0.047)	0.455 *** (0.054)	0.403 *** (0.053)
Age		−0.006 (0.039)		−0.007 (0.044)
Gender		0.138 (0.283)		0.154 (0.315)
Marriage		0.089 ** (0.041)		0.099 ** (0.045)
Children’s number		0.009 (0.086)		0.009 (0.096)
Residence		−0.114 ** (0.058)		−0.127 ** (0.065)
Household registration		−0.056 (0.132)		−0.063 (0.147)
Education level		0.055 (0.093)		0.062 (0.104)
Household income level		0.000 (0.005)		0.001 (0.005)
Social security		−0.006 * (0.002)		−0.007 * (0.002)
Economic supply		0.000 (0.000)		0.000 (0.000)
Internet usage		0.087 * (0.010)		0.096 * (0.011)
Individual fixed-effects	Yes	Yes	Yes	Yes
Year fixed-effects	Yes	Yes	Yes	Yes
Observations	14,196	14,196	14,196	14,196
R2	0.030	0.055	0.035	0.059

Note: Robust standard errors in parentheses. * *p* < 0.1, ** *p* < 0.05, *** *p* < 0.01. All models include individual fixed-effects, year fixed-effects, and control variables.

**Table 3:** PSM-DID balance test results.

Variables		Sample Mean		Standard Deviation (%)	T-Test	
		Test Group	Control Group		T-Value	*p*-Value
Age	Unmatched	70.70	70.75	−0.6	−0.17	0.864
	Matched	70.70	70.31	5.8	1.11	0.266
Gender	Unmatched	0.464	0.508	−8.7	−2.35	0.019 **
	Matched	0.465	0.464	0.3	0.05	0.960
Marriage	Unmatched	0.773	0.737	8.3	2.22	0.027 **
	Matched	0.774	0.768	1.5	0.30	0.764
Children’s number	Unmatched	1.809	2.422	−50.6	−13.00	0.000 ***
	Matched	1.808	1.750	4.8	1.07	0.284
Residence	Unmatched	0.941	0.890	18.5	4.51	0.000 ***
	Matched	0.941	0.918	8.3	1.78	0.075 *
Household registration	Unmatched	0.821	0.410	93.2	22.99	0.000 ***
	Matched	0.821	0.847	−5.8	−1.36	0.175
Education level	Unmatched	3.719	2.930	59.9	16.16	0.000 ***
	Matched	3.720	3.741	−1.6	−0.32	0.750
Household income level	Unmatched	10.76	10.34	40.8	8.91	0.000 ***
	Matched	10.76	10.72	4.1	1.06	0.288
Social security	Unmatched	7.360	5.487	71.4	18.53	0.000 ***
	Matched	7.357	7.290	2.5	0.51	0.610
Economic supply	Unmatched	5090	3159	38.6	13.23	0.000 ***
	Matched	5066	4498	11.3	2.02	0.044 **
Internet usage	Unmatched	3.288	1.751	89.3	27.54	0.000 ***
	Matched	3.286	3.415	−7.5	−1.35	0.176

Note: * *p* < 0.1, ** *p* < 0.05, *** *p* < 0.01.

**Table 4:** Replacement of the explained variable.

Variables	(1) Social Adaptability	(2) Social Adaptability Inequality
Smart wearable devices	0.081 ** (0.040)	0.127 ** (0.060)
Controlled variables	Yes	Yes
Individual fixed-effects	Yes	Yes
Year fixed-effects	Yes	Yes
Observations	14,196	14,196
R^2^	0.016	0.015

Note: Robust standard errors in parentheses. ** *p* < 0.05. All models include individual fixed-effects, year fixed-effects, and control variables.

**Table 5:** Instrumental variable regression results.

Variables	(1) Health	(2) Health Inequality
Smart wearable devices	2.297 *** (3.70)	2.557 *** (3.70)
Controlled variables	Yes	Yes
Individual fixed-effects	Yes	Yes
Year fixed-effects	Yes	Yes
Cragg–Donald Wald F Statistic	73.362 ***	73.362 ***
Kleibergen–Paap rk LM Statistic	74.801 ***	74.801 ***
Observations	14,196	14,196

Note: Robust t-statistics in parentheses. *** *p* < 0.01. All models include individual fixed-effects, year fixed-effects, and control variables.

**Table 6:** Mediation analysis results.

Variables	(1) Health	(2) Health	(3) Health Inequality	(4) Health Inequality
Smart wearable devices	0.338 * (0.048)	0.359 * (0.047)	0.376 * (0.053)	0.399 * (0.052)
Joy of living	0.088 * (0.018)		0.098 * (0.020)	
Social participation		0.009 (0.004)		0.010 (0.004)
Controlled variables	Yes	Yes	Yes	Yes
Individual fixed-effects	Yes	Yes	Yes	Yes
Year fixed-effects	Yes	Yes	Yes	Yes
Observations	14,196	14,196	14,196	14,196
R^2^	0.061	0.056	0.065	0.061

Note: Robust standard errors in parentheses. * *p* < 0.1. All models include individual fixed-effects, year fixed-effects, and control variables.

**Table 7:** Heterogeneity analysis results.

Variables		(1) Health	(2) Health Inequality
Income	Low-income	−0.049 (0.110)	−0.054 (0.122)
	High-income	0.446 *** (0.045)	0.495 *** (0.050)
Education	Low education level	0.372 *** (0.035)	0.413 *** (0.039)
	High education level	0.225 ** (0.072)	0.249 ** (0.080)
Urban-rural division	Rural area	0.483 *** (0.076)	0.537 *** (0.084)
	Urban area	0.341 *** (0.038)	0.379 *** (0.042)
Gender	Female	0.320 *** (0.044)	0.355 *** (0.049)
	Male	0.357 *** (0.046)	0.397 *** (0.051)
Controlled variables		Yes	Yes
Individual fixed-effects		Yes	Yes
Year fixed-effects		Yes	Yes
Observations		14,196	14,196

Note: The coefficients in the table are regression results for smart wearable devices; standard errors are in parentheses; ** *p* < 0.05, *** *p* < 0.01. All models include individual fixed-effects, year fixed-effects, and control variables.

## Data Availability

CLASS data are available at http://class.ruc.edu.cn/English/Home.htm (accessed on 15 October 2024).
